# Phenotypic Plasticity of the Introduced New Zealand Mud Snail, *Potamopyrgus antipodarum*, Compared to Sympatric Native Snails

**DOI:** 10.1371/journal.pone.0093985

**Published:** 2014-04-03

**Authors:** Edward P. Levri, Amy C. Krist, Rachel Bilka, Mark F. Dybdahl

**Affiliations:** 1 Division of Math and Sciences, Penn State – Altoona, Altoona, Pennsylvania, United States of America; 2 Department of Zoology & Physiology and Program in Ecology, University of Wyoming, Laramie, Wyoming, United States of America; 3 Department of Biological Sciences, Washington State University, Pullman, Washington, United States of America; National University of Singapore, United States of America

## Abstract

Phenotypic plasticity is likely to be important in determining the invasive potential of a species, especially if invasive species show greater plasticity or tolerance compared to sympatric native species. Here in two separate experiments we compare reaction norms in response to two environmental variables of two clones of the New Zealand mud snail, *Potamopyrgus antipodarum*, isolated from the United States, (one invasive and one not yet invasive) with those of two species of native snails that are sympatric with the invader, *Fossaria bulimoides* group and *Physella gyrina* group. We placed juvenile snails in environments with high and low conductivity (300 and 800 mS) in one experiment, and raised them at two different temperatures (16°C and 22°C) in a second experiment. Growth rate and mortality were measured over the course of 8 weeks. Mortality rates were higher in the native snails compared to *P. antipodarum* across all treatments, and variation in conductivity influenced mortality. In both experiments, reaction norms did not vary significantly between species. There was little evidence that the success of the introduced species is a result of greater phenotypic plasticity to these variables compared to the sympatric native species.

## Introduction

Phenotypic plasticity has long been thought to underlie the ability of a species to colonize new environments or communities and become invasive (i.e., geographically widespread and ecologically dominant) [1]. There have been two primary approaches to studying phenotypic plasticity in invasives: 1) comparing the reaction norms of invasive populations to non-invasive populations of the same species to demonstrate the evolution of phenotypic plasticity as an adaptive trait for invasions, and 2) comparing the phenotypic plasticity of invasive populations to competing native populations [2]. Demonstrating greater phenotypic plasticity in invaders generally suggests that phenotypic plasticity is a preadaptation for invasion success. A large number of studies have measured the plasticity of invasive plants relative to non-invasive or native species, generally finding that invasive species have greater plasticity (reviewed in [2]). Richards et al. [2] proposed several hypotheses about the form of fitness-related phenotypic plasticity in successful invasive species compared to native species. Relative to native species, phenotypic plasticity of invasive species may facilitate invasion success by maintaining high fitness despite stressful conditions, achieving higher fitness in favorable conditions, or permitting higher fitness or tolerance under a broader set of conditions [2]. Under each of these possible reaction norms (forms of plasticity), invasive organisms maintain higher mean fitness than natives.

The New Zealand mud snail, *Potamopyrgus antipodarum*, has been introduced in the United States [3], [4], [5] and around the world [6]. Phenotypic plasticity may play a role in the environments where *P. antipodarum* is invasive. The snail is known to behave differently in the presence and absence of fish predators [7] and snails from different populations respond to fish predators differently [8]. Significant variation also exists between invasive and native clones in their growth rates at various salinities with invasive clones maintaining greater fitness at higher salinities [9]. Also, Dybdahl and Kane [10] demonstrated significant plasticity in fitness across temperatures. Other experiments have indicated that shell shape is in part phenotypically plastic in invasive populations and may be important in conforming to stream flow variation [11]. Directly and indirectly, these studies suggest that multiple traits in *P. antipodarum* demonstrate phenotypic plasticity, and some studies show that invasive populations appear more plastic than populations of *P. antipodarum* from the native range (New Zealand). However, whether invasion success results from greater plasticity of *P. antipodarum* compared to native species has not been tested in this system.

At least two different clones of the New Zealand mud snail currently exist in rivers and streams of the western US where they have been established since at least 1987 [12]. One clone (US1) is invasive, inhabiting all of the western US states except for New Mexico and causes ecological disruption [13], [14], while another clone is not apparently invasive (US3), with a restricted distribution in the Snake River in Idaho where its range has not expanded significantly since its identification around 2005 [15]. In the Greater Yellowstone Area, the invasive clone (US1) is sympatric with two native pulmonate snails in many streams and can occur on the same rocks and submerged macrophytes (Krist, unpublished data). The native snail *Physella (Physella) gyrina* group (Physidae) is widespread in the Greater Yellowstone Area whereas the distribution of the other native *Fossaria (Bakerilymnaea) bulimoides* group (Lymnaeidae) is more restricted (Krist, unpublished data). The ecological similarity of the introduced and native snails is also exhibited by their diets. *P. antipodarum* consumes primarily periphyton (organic biofilm on rocks and vegetation) [16], [17] and fine organic matter [16], and both *Fossaria* (Thon and Krist, unpublished data) and *Physella* (reviewed in [18]) are known to consume periphyton.

In two experiments, we addressed whether differences in phenotypic plasticity between the non-native *P. antipodarum* and two native species in response to conductivity and temperature may have contributed to the invasion success of *P. antipodarum*. We chose conductivity and temperature because both abiotic factors affect growth and survival of New Zealand mud snails [10], [19], and temperature also affects reproduction [10]. Specifically, we compared reaction norms (the range of phenotypes an individual exhibits across environments) of growth rate and survival between two clones, one invasive (US1) and one non-invasive (US3) with the reaction norms of the two native snails. If phenotypic plasticity in response to conductivity or temperature contribute to the invasion success of the successful invasive clone, then we expect the reaction norm for fitness-related traits of the invasive clone to differ from the non-invasive clone and probably from the two native species as well, contributing to greater tolerance to stressful or more variable conditions [2].

## Methods

In June 2010, we collected the US1 clone of *P. antipodarum*, the widespread invasive, from Polecat Creek in the Rockefeller National Parkway, WY and the two native snail species, *Fossaria (Bakerilymnaea) bulimoides* group and *Physella (Physella) gyrina* group, from ∼3 km upstream in an unnamed tributary of Polecat Creek. US3, the non-invasive clone, was originally collected from the Snake River. None of the snails that we used in our experiments are an endangered or protected species, and necessary permits were granted by Grand Teton National Park (United States National Park Service). The experiments were conducted at the Red Buttes Environmental Laboratory at the University of Wyoming.

We conducted two experiments, one where snails of each type were exposed to two different conductivities and one where the snails were exposed to two different temperatures. For each treatment of each experiment, we housed twenty juvenile snails of each *P. antipodarum* clone (US1 and US3) and the two native snails (*Fossaria and Physella*) individually in 100 ml glass beakers for a total of 80 juveniles of each snail type (two clones of *P. antipodarum, Physella*, and *Fossaria*). Snails were housed singly in a 100 ml beaker and fed 0.96 mg of *Spirulina* powder (Argent Laboratories) three times per week. Thus twenty snails of each type were exposed to each treatment in each experiment. Snails were housed under controlled light (12∶12-h light∶dark cycle). We measured shell length using an ocular micrometer in a dissecting microscope by noting the distance from the apex of the shell to the furthest edge of the aperture when the aperture of the snail was facing up (0.1 mm resolution). The initial size ranges of the juvenile snails were 1.04–2.02 mm for US1, 1.23–2.20 mm for US3, 3.17–4.76 mm for *Fossaria*, and 2.56–4.59 mm for *Physella*. New Zealand mud snails very rarely become reproductively mature at <2.5 mm in shell length [20]. For *Fossaria*, we used the same size range as we had used in another experiment measuring *Fossaria* growth (Thon et al. in prep.). For *Physella*, we collected the smallest ∼20% of individuals at the study site to ensure that we included only juvenile individuals. The initial size distributions for US1 and US3 were compared using ANOVA and were not significantly different. At the beginning of the experiment and weekly thereafter for eight weeks, we measured shell length under a dissecting microscope. Three times a week we changed water in the beakers and noted all mortality. We were confident that we could assess growth rate in all species because the duration of our experiments, eight weeks, greatly exceeded previous studies where we have measured growth in *P. antipodarum* (e.g. [21] 18 days) and in *Fossaria* (Thon et al. unpublished data; 21 days).

In the conductivity experiment we produced low (300 μS) and high (800 μS) conductivity levels because this range is typical of normal levels in freshwater habitats where these species exist (http://www.kimberly.uidaho.edu/midsnake/ ; Dybdahl et al. unpublished data). We used spring water (pH = 7.85, 192 ppm CaCO_3_) with conductivity of 800 μS to obtain high conductivity, and we diluted the spring water with distilled water to obtain the low conductivity treatment of 300 μS.

In the temperature experiment, we used two different temperatures (16°C, 22°C) with 16°C being the year round temperature where the US1 clone was collected (Riley and Dybdahl, unpublished data) and 22°C known to be relatively stressful [10]. Previous work suggests that 16°–18°C are favorable temperatures for *P. antipodarum* [10]. We also expect the higher temperature to reflect the effects of climate change as reduced precipitation in the Greater Yellowstone Area [22] should increase temperatures in streams and other surface waters. We controlled water temperature by immersing the beakers housing the snails in thermostat-controlled water baths (Boekel Industries Inc., Model 148007).

All statistical analyses were performed using IBM SPSS Statistics v. 20. We analyzed survival rates in both experiments using log-linear analysis with a backwards elimination procedure with proportion surviving as the dependent variable and snail type and treatment as independent variables. We first used a fully factorial model and then used planned contrasts to compare the survival rate of the US1 clone of *P. antipodarum* to US 3 and the two native species for both conductivity and temperature. For the planned contrasts, we adjusted the critical P-value to 0.017 to accommodate the three additional contrasts per analysis. We planned contrasts to the US1 clone because it is the invasive clone. Specific growth rates (SGR) were calculated for each snail type in each treatments using the formula SGR  =  ln(final length/initial length)/days of experiment. To compare specific growth rates in each experiment we utilized two-way ANOVA with snail type and treatment as independent variables. We used a fully factorial model and then compared each snail type to the US1 clone of *P. antipodarum* using post-hoc comparisons with Tukey's honest significant differences. If the homogeneity of variances assumption was violated a weighted ANOVA was performed by weighting the data using the inverse square root of the variances of each factor group [23].

## Results

**Figure 1:**
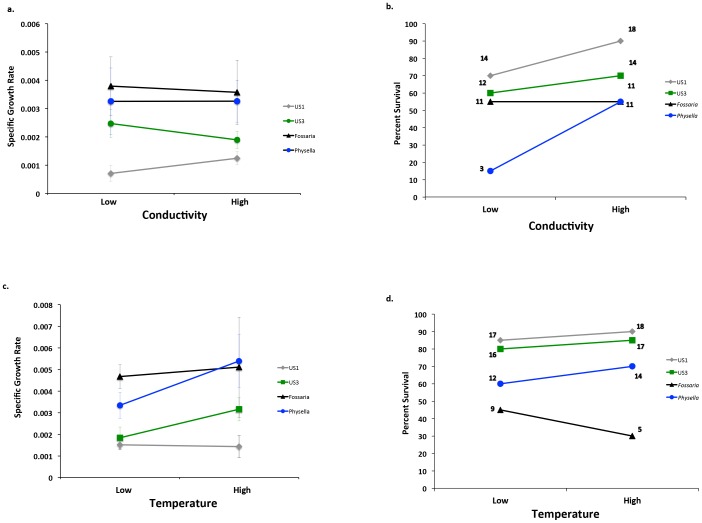
The effect of variation in conductivity on snail specific growth rate (a) and mortality (b) and the effect of variation in temperature on the specific growth rate (c) and mortality (d). Error bars for specific growth rates are standard errors of the mean. Numbers on the mortality figures indicate the number of surviving snails out of twenty.

Conductivity: The fully factorial model revealed a significant effect of snail type on specific growth rates (Table I) with the *P. antipodarum* clones generally growing slower than the two native species (Figure 1a). However, we found no evidence of effects of the conductivity treatments or a conductivity by snail type interaction. When specifically comparing the US1 and US3 clones, the US3 clone had a significantly higher specific growth rate than the US1 clone (Table I; Figure 1a). Both native snails grew at a significantly greater rate than the *Potamopyrgus* US1 clone (Table I).

**Table 1:** Results of Univariate ANOVA comparing the specific growth rate of two clones of *P. antipodarum* and two native snails in the conductivity experiment.

Source	df	F	P
Intercept	1	117.37	<0.0005
Snail type	3	9.692	<0.0005
Treatment	1	0.019	= 0.892
Snail type x Treatment	3	0.638	= 0.592
Error	54		

Tukey HSD	Mean Diff.	Std. Err.	P
US1 vs US3	−0.0011	0.0004	= 0.040
US1 vs *Fossaria*	−0.0027	0.0006	<0.0005
US1 vs *Physella*	−0.0022	0.0006	= 0.002

A post hoc Tukey's honest significant differences procedure was performed to specifically compare the specific growth rates of the invasive US1 clone to each other snail type.

For survival, the fully factorial model showed effects of snail type (X^2^ = 18.207, d.f. = 1, P<0.0005) and conductivity treatment on survival (X^2^ = 5.085, d.f. = 1, P = 0.024), but no significant treatment by snail type interaction (X^2^ = 4.662, d.f. = 3, P = 0.198) (Figure 1b). When comparing US1 to US3, the survival rate of the two introduced clones of *P. antipodarum* was not influenced by conductivity (X^2^ = 2.279, d.f. = 1, P = 0.131), nor was there a significant three-way interaction between snail type, survival and conductivity treatment (X^2^ = 0.688, d.f. = 1, P = 0.407). Thus we detected no difference in phenotypic plasticity between US1 and US3 in response to conductivity. Comparing US1 to *Fossaria*, the US1 clone survived at a significantly greater rate overall (X^2^ = 5.81, d.f. = 1, P = 0.016 [P_crit_ = 0.017]), but there was no effect of conductivity on survival (X^2^ = 0.985, df = 1, P = 0.321 [P_crit_ = 0.017]) nor a conductivity by snail type interaction (X^2^ = 1.609, df = 1, P = 0.205 [P_crit_ = 0.017]). Similarly, when comparing the US1 clone to *Physella*, we found that the US1 clone survived at a significantly greater rate overall (X^2^ = 17.27, df = 1, P<0.0005 [P_crit_ = 0.017]), and the higher conductivities increased survival (X^2^ = 7.495, df = 1, P = 0.006 [P_crit_ = 0.017]). However there was no significant treatment by snail type interaction (X^2^ = 0.246, df = 1, P = 0.620 [P_crit_ = 0.017]).

Temperature: The fully factorial model indicated a significant effect of snail type on the specific growth rate, but we found no effect of temperature treatment nor a snail by treatment interaction (Table II). Specific comparisons showed no difference in growth rates between the US1 and US3 clones (Table II; Figure 1c), and both native snails grew at a greater rate than the US1 clone (Table II).

**Table 2:** Results of Univariate ANOVA comparing the specific growth rate of two clones of *P. antipodarum* and two native snails in the temperature experiment.

Source	df	F	P
Intercept	1	144.46	<0.0005
Snail type	3	10.257	<0.0005
Treatment	1	2.865	= 0.094
Snail type x Treatment	3	1.230	= 0.303
Error	54		

Tukey HSD	Mean Diff.	Std. Err.	P
US1 vs US3	−0.0010	0.0005	= 0.019
US1 vs *Fossaria*	−0.0033	0.0007	<0.0005
US1 vs *Physella*	−0.0026	0.0006	<0.0005

A post hoc Tukey's honest significant differences procedure was performed to specifically compare the specific growth rates of the invasive US1 clone to each other snail type.

The different snail types survived at different rates according to a fully factorial model (X^2^ = 30.955, df = 3, P<0.0005). In general, the *P. antipodarum* clones survived better than the two native species (Figure 1d). However, the temperature treatment had little effect on survival (X^2^ = 0.001, df = 1, P = 1.00), and we did not detect a temperature by snail type interaction (X^2^ = 0.001, df = 3, P = 1.00). The US1 and US3 clones did not differ in survival (X^2^ = 0.394, df = 1, P = 0.530 [P_crit_ = 0.017]), nor did they differ in survival in response to the temperature treatments (X^2^ = 0.396, df = 1, P = 0.529 [P_crit_ = 0.017]), nor was there any evidence of a genotype by environment interaction (X^2^ = 0.008, df = 1, P = 0.929 [P_crit_ = 0.017]). Comparisons of the US1 clone to both native snails were very similar: US1 had a significantly greater survival rate than both native snails (*Fossaria*, X^2^ = 24.88, df = 1, P<0.0005 [P_crit_ = 0.017]; *Physella*, X^2^ = 5.77, df = 1, P = 0.016 [P_crit_ = 0.017]) but the temperature treatments did not affect survival of any of the snails (*Fossaria*, X^2^ = 0.475, df = 1, P = 0.491 [P_crit_ = 0.017]; *Physella*, X^2^ = 0.623, df = 1, P = 0.430 [P_crit_ = 0.017]) nor was there a significant temperature by snail type interaction for either comparison (*Fossaria*, X^2^ = 1.337, df = 1, P = 0.248 [P_crit_ = 0.017]; *Physella*, X^2^ = 0.001, df = 1, P = 0.985 [P_crit_ = 0.017]).

## Discussion

Conductivity and temperature influence the distribution and abundance of gastropods [24], so we exposed introduced, invasive and native snails to different levels of these two environmental variables to determine whether phenotypic plasticity contributes to invasion success. We found no evidence for differences in phenotypic plasticity among two clones of the introduced snail *P. antipodarum* and two native snail species. The relatively parallel reaction norms for snail growth and survival (Figure 1), as demonstrated by the non-significant snail type by treatment interaction effects, suggest that both the invasive and the more restricted clones of the introduced species and the two native snails did not differ in their response to our experimental levels of conductivity and temperature.

As expected, we found a significant effect of conductivity level on survival, but surprisingly, not on growth. Conductivity is a measure of the concentration of ions and is known to influence the distribution and composition of snail communities (e.g. [25], [26]). At low conductivities, important ions crucial for snail growth and survival (e.g. calcium) are too dilute, and high conductivities can cause stressful osmotic pressure. Despite the significantly lower survival at low conductivity across the introduced, invasive, and native snails, all snail types responded similarly. The absence of a significant effect of conductivity on growth rates might indicate that the influence of conductivity is greatest on survival, and that the growth of surviving individuals was tolerant of low conductivity.

Surprisingly, all snail types responded similarly to experimental temperature conditions. Although *P. antipodarum* can survive temperatures as low as 4°C and as high as 32°C [31], temperature appears to be a limiting factor because populations of *P. antipodarum* exhibit winter population crashes [3], [27], [28], [29]. Lab experiments showed that temperature has a strong effect on fitness [10], [30]; the invasive clone US1 grows and reproduces best at intermediate temperatures around 18°C, and these traits respond strongly to variation around that optimum [10]. Consequently, our temperature treatments should have induced responses in growth or survival in these snail types. Nevertheless, the introduced, invasive, and native snails responded similarly to our temperature treatments, as demonstrated by the non-significant differences in reactions norms among them.

We expected the successfully invasive clone of *P. antipodarum* (US1) to possess a form of phenotypic plasticity that is either robust to harsh conditions, more opportunistic under good conditions, or better under all conditions [2]. First, we expected that the response to temperature or conductivity of the invasive US1 clone of *P. antipodarum* to differ from the US3 clone, which is introduced but restricted in its distribution. While the flat reaction norms (Figure 1) are consistent with the capacity of US1 to maintain higher fitness across experimental temperature or conductivity conditions, we found that US3 also possessed the same reaction norm. In fact, US3 was better under all conditions compared to US1; US3 either grew at the same rate (temperature experiment) or faster (conductivity experiment) than US1. Second, we also expected US1 to possess a different form of phenotypic plasticity than the two native species. For survival, both US1 and US3 survived at higher rates in both experiments than the native species, showing greater fitness under all conditions. For growth rate, *P. antipodarum* and the two native species might not be directly comparable because of differences in growth stage of experimental snails and size at maturity. However, it is possible to compare directly the reaction norms of the different species, yet we detected no differences between the reaction norms of natives and non-natives in either experiment. These results, taken together, indicate little difference in the reaction norms among introduced, invasive and native snails under experimental temperature and conductivities.

Our goal was to expose snails to realistic environmental conditions to determine whether phenotypic plasticity plays a role in the success of an invasive genotype compared to a non-invasive genotype or native species. We did not find any evidence for significant variation among invasive, non-invasive, and native snails in the reaction norms under our experimental conductivity and temperature variation. It is possible that invasion success may be facilitated by an individual's ability to cope with a temporally changing environment, which we did not address since we maintained constant temperature and conductivity conditions in our treatments. Furthermore, the possibility exists that invasive *P. antipodarum* may demonstrate phenotypic plasticity with respect to other traits or abiotic or biotic variables not measured here that may be important in its invasion success. For example, the US1 genotypes differ in shell shape across populations, and shell shape represents a plastic response to stream water velocities [11]. This response, if different from those of native snails, might result in fitness advantages that help to explain invasion success.
